# TRIB3 inhibits proliferation and promotes osteogenesis in hBMSCs by regulating the ERK1/2 signaling pathway

**DOI:** 10.1038/s41598-017-10601-w

**Published:** 2017-09-04

**Authors:** Cui Zhang, Fan-Fan Hong, Cui-Cui Wang, Liang Li, Jian-Ling Chen, Fei Liu, Ren-Fu Quan, Jin-Fu Wang

**Affiliations:** 1 Institute of Cell and Development Biology, College of Life Sciences, Zijingang Campus, Zhejiang University, Hangzhou, Zhejiang, 310058 P. R. China; 2 Institute of Orthopedics, Xiaoshan Traditional Chinese Medical Hospital, Hangzhou, Zhejiang, 311200 P. R. China

## Abstract

Osteogenic differentiation in human bone marrow-derived mesenchymal stem cells (hBMSCs) is regulated by various factors, including bone morphogenetic proteins (BMPs), Notch, growth hormones and mitogen-activated protein kinases (MAPKs). Tribbles homolog 3 (TRIB3), a pseudokinase, plays an important role in cancer cells and adipocytes. However, TRIB3 function in osteogenic differentiation is unknown, although it is involved in regulating signaling pathways associated with osteogenic differentiation. Here, we found that TRIB3 was highly expressed during osteogenic differentiation in hBMSCs. Inhibition of focal adhesion kinase (FAK) or phosphatidylinositol 3-kinase (PI3K) resulted in a significant decrease in TRIB3 expression, and expression of TRIB3 was restored by increasing insulin-like growth factor-1 (IGF-1) via activating phosphatidylinositol 3-kinase/protein kinase B (PI3K/AKT) signaling. TRIB3 knock-down enhanced proliferation and decreased osteogenic differentiation at the middle stage of differentiation, and these effects were reversed by inhibiting the activation of extracellular signal-regulated kinase (ERK)-1/2. In conclusion, TRIB3 plays an important role in proliferation and osteogenic differentiation by regulating ERK1/2 activity at the middle stage of differentiation, and expression of TRIB3 is regulated by FAK in a PI3K/AKT-dependent manner.

## Introduction

Human bone marrow-derived mesenchymal stem cells (hBMSCs) have the potential to differentiate into various lineages of mesenchymal tissues, such as bone, cartilage, fat, muscle, and marrow stroma^1^. hBMSCs have been used as a promising cell source for the regeneration of tissues, including bone, in several clinical trials^2, 3^. Migration and osteogenic differentiation in mesenchymal stem cells (MSCs) play a critical role in the partial coalescence of fractures^4–6^. Osteogenic differentiation in MSCs is regulated by many factors, such as bone morphogenetic proteins (BMPs)^7^, growth hormones (GHs)^8^, mitogen-activated protein kinases (MAPKs)^9, 10^, and Hedgehog^11, 12^. In addition, the Notch^13^ and Wnt^14, 15^ signaling pathways also play a regulatory role in osteogenic differentiation in MSCs.

Tribbles homolog 3 (TRIB3), a mammalian homolog of Drosophila tribbles, is a pseudokinase with a kinase domain that lacks enzyme activity^16^. TRIB3 is expressed in various tissues, such as liver^17^, adipose^18^, heart^19^, and skeletal muscles^20^. Studies suggest that TRIB3 performs strikingly different metabolic functions and has been demonstrated to interact with several transcriptional mediators. TRIB3 has been identified as a negative regulator of protein kinase B (AKT) activity and participates in insulin signaling in HEK293 cells and liver. TRIB3 disrupts insulin signaling by binding directly to AKT and inhibits the insulin-stimulated AKT phosphorylation of Thr308 and Ser473; TRIB3 also contributes to insulin resistance in individuals who are susceptible to type II diabetes^17^. Likewise, the overexpression of TRIB3 in C2C12 myoblasts significantly reduces insulin-stimulated AKT phosphorylation^20^. TRIB3 also negatively regulates adipogenesis by blocking the CCAAT/enhancer-binding protein β (C/EBP β) proadipogenic function^21^. A recent study has shown that TRIB3 is also involved in apoptosis. The C/EBP homologous protein/activating transcription factor 4 (CHOP/ATF4) pathway can induce TRIB3 expression, and the knock-down of TRIB3 expression decreases ER stress-dependent cell death^22^. In addition, TRIB3 is also involved in the canonical transforming growth factor-β (TGF-β), BMPs, MAPKs, nuclear factor-kappaB (NF-κB), and Notch signaling pathways. In tumor cells, TRIB3 augments TGF-β1-SMAD3-mediated transcriptional activity and cellular functions by physically interacting with SMAD2/3. Knock-down of TRIB3 expression in tumor cells significantly inhibits the invasive and metastatic ability of the cells by promoting the mesenchymal-epithelial transition^23^. TRIB3 plays an important role in fibroblast activation in systemic sclerosis (SSc) by activating the canonical TGF-β/SMAD signaling pathway and stimulating the release of collagen, thereby inducing a positive feedback loop that may contribute to aberrant TGF-β signaling in SSc^24^. In addition, TRIB3 is known as a master regulator of Notch via the MAPK-ERK and TGF-β pathways and is required for the growth of basal-like breast cancer^25^. These signaling pathways are also associated with the regulation of osteogenic differentiation, but the function of TRIB3 in osteogenic differentiation is unknown.

Here, we investigated the role of TRIB3 in osteogenic differentiation in hBMSCs. Our results showed that TRIB3 was overexpressed in osteogenically induced cells and that TRIB3 expression was regulated by focal adhesion kinase (FAK) activity in a phosphatidylinositol 3-kinase/protein kinase B (PI3K/AKT)-dependent manner. TRIB3 inhibited cell proliferation and promoted osteogenic differentiation in hBMSCs by inhibiting the activity of extracellular signal-regulated kinase (ERK)-1/2 at the middle stage of osteogenesis.

## Results

### The effects of TRIB3 on osteogenic differentiation in hBMSCs

**Figure 1:**
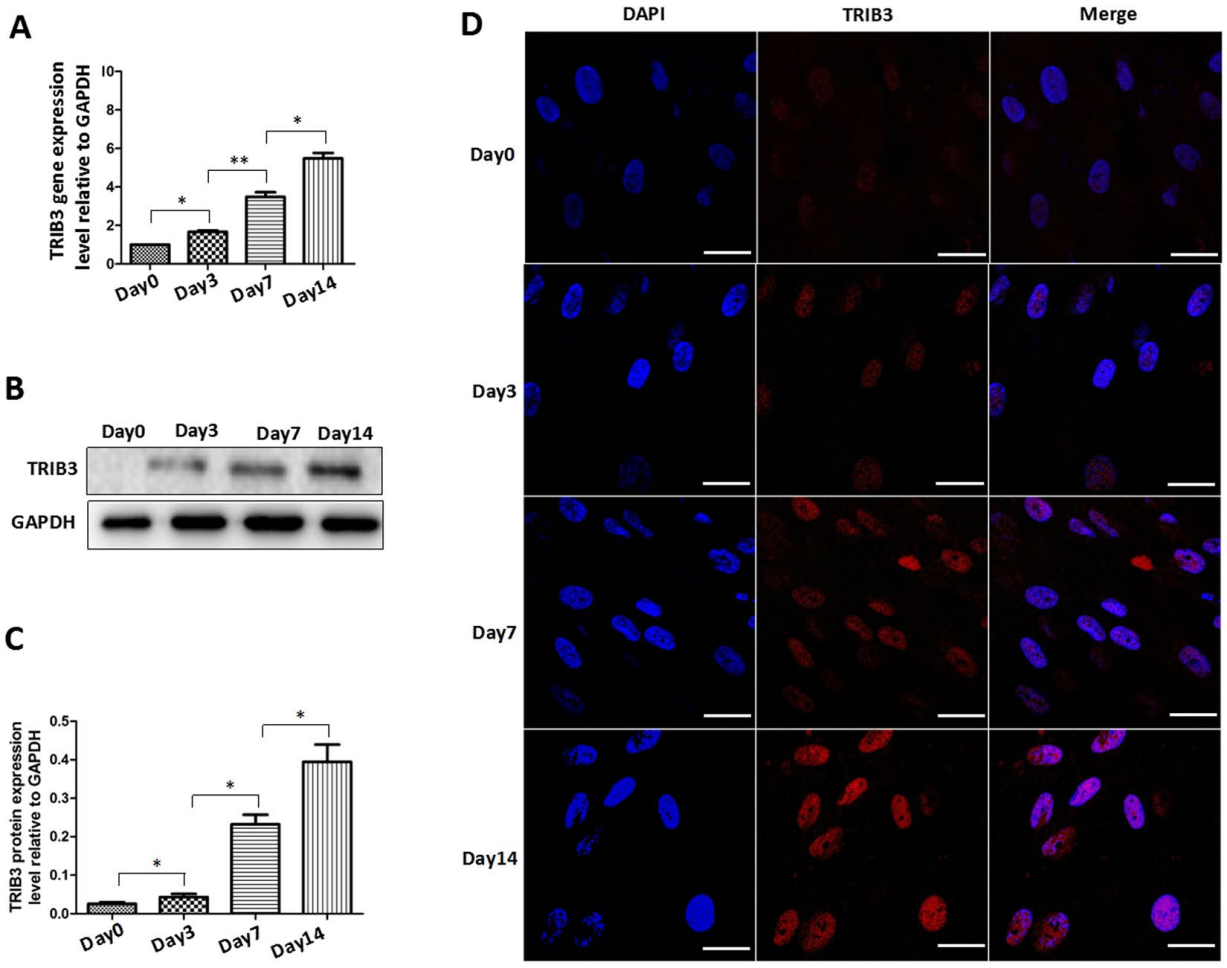
Expression of TRIB3 during the osteogenic differentiation of hBMSCs. (**A**) Real-time PCR analysis of TRIB3 mRNA expression in cells induced for 0, 3, 7, and 14 days. (**B**) Western blot analysis of TRIB3 protein expression in cells induced for 0, 3, 7, and 14 days. (**C**) Relative expression level of TRIB3 protein quantified and plotted. (**D**) Expression of TRIB3 determined by immunostaining with anti-TRIB3 at days 0, 3, 7, and 14 of osteogenic differentiation. Red: TRIB3; Blue: DAPI. Scale: 40 μm. *P < 0.05, **P < 0.01.

To investigate the role of TRIB3 in osteogenic differentiation in hBMSCs, we first analyzed the TRIB3 mRNA and protein levels at the early stage (day 0 and day 3) and middle stage (day 7 and day 14) of osteogenic differentiation by real-time RT-PCR, western blot and immunofluorescence. As shown in Figs 1A–C and S2, TRIB3 mRNA and protein levels increased from days 0 to 14 and reached their peak at day 14 of differentiation. Immunofluorescent staining revealed that the fluorescence intensity increased with the increase in the induction time (Fig. 1D) and that TRIB3 was located in the nucleus. These results indicate that TRIB3 is expressed throughout osteogenic differentiation and its expression level increases with the induction time, suggesting an important role for TRIB3 in osteogenic differentiation in hBMSCs.

**Figure 2:**
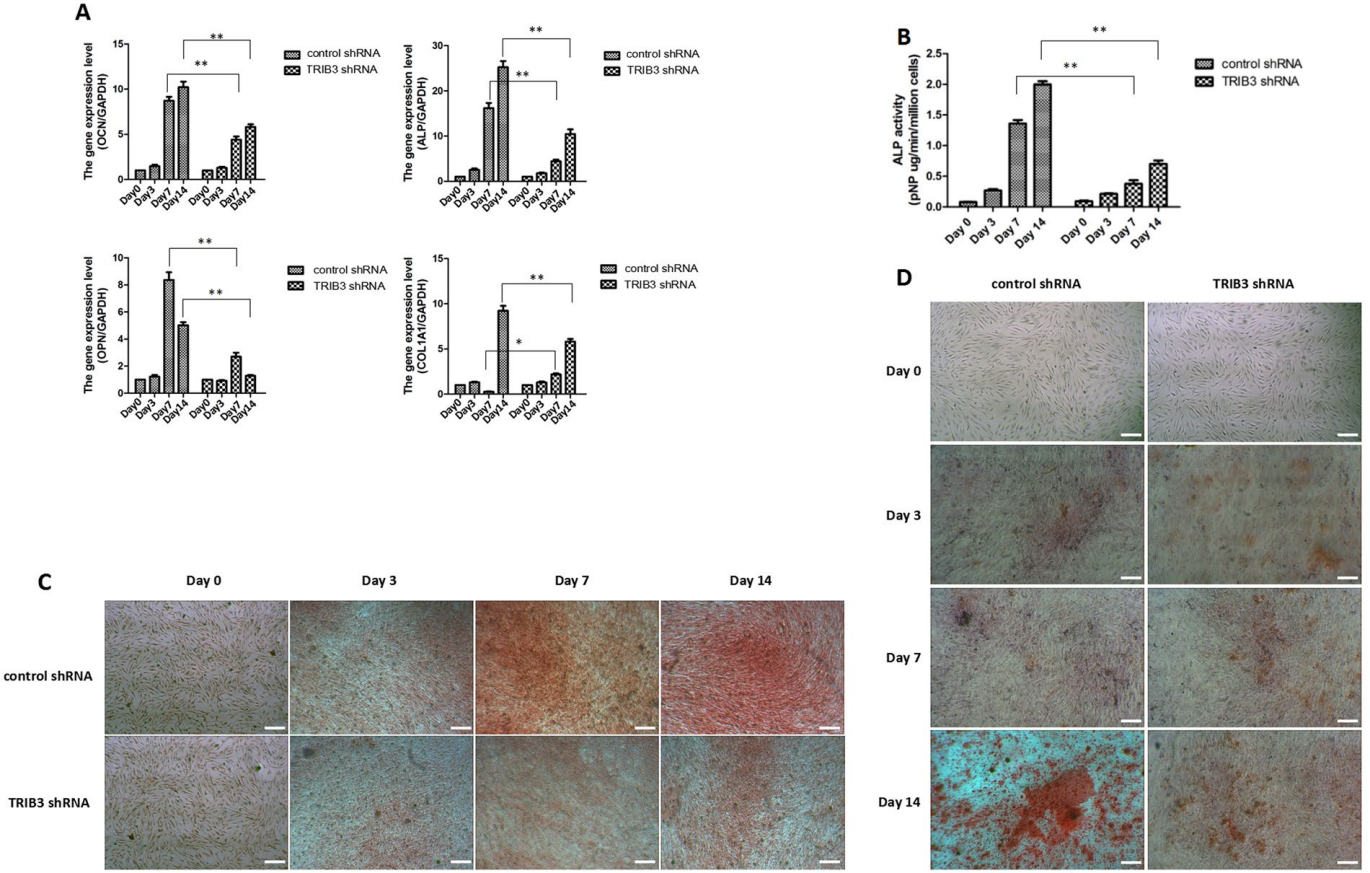
Effects of TRIB3 knock-down on the osteogenic differentiation of hBMSCs. (**A**) Real-time PCR analysis of marker genes specific for osteoblasts, such as *ALP*, *COL1A1*, *OPN*, and *OCN*, in cells induced for 0, 3, 7, and 14 days. (**B**) Analysis of ALP activity in cells induced for 0, 3, 7 and 14 days. (**C**) ALP staining of cells induced for 0, 3, 7 and 14 days. Scale: 100 μm. (**D**) Mineralization (intense red clusters) in cells induced for 0, 3, 7 and 14 days. Scale: 100 μm. control shRNA: hBMSCs transfected with negative control-shRNA-Vector; TRIB3 shRNA: hBMSCs transfected with TRIB3-shRNA-Vector. *P < 0.05, **P < 0.01.

Then, we knocked down TRIB3 via transfection with TRIB3-shRNA-Vector (TRIB3 shRNA) and performed real-time PCR, immunoblotting and immunofluorescence assays to detect the expression of TRIB3 mRNA and protein and evaluate the efficiency of TRIB3 knock-down (Fig. S2). The potential for osteogenesis was evaluated by measuring the mRNA expression of four osteogenic markers - alkaline phosphatase (ALP), collagen type 1 alpha 1 chain (COL1A1), osteopontin (OPN), and osteocalcin (OCN). As shown in Fig. 2A, TRIB3 knock-down inhibited the expression of all osteogenic markers at the middle stage of osteogenesis but did not affect that at the early stage. To confirm the effects of TRIB3 knock-down on the differentiation of hBMSCs into osteoblasts, we used a functional assay to quantify ALP activity and mineralization. The cells induced from hBMSCs and transfected with TRIB3-shRNA-Vector showed a decrease in ALP activity at day 7 and day 14 of induction, but no change in ALP activity at day 0 and day 3 of induction, compared to that in cells induced from hBMSCs and transfected with negative control-shRNA-Vector (Fig. 2B and C). Furthermore, we used ARS assays to analyze the role of TRIB3 in the induction of mineralization. In comparison to cells induced from hBMSCs and transfected with negative control-shRNA-Vector, cells induced from hBMSCs and transfected with TRIB3-shRNA-Vector showed less mineralized nodules at day 14 (Fig. 2D). Induction for 0, 3 and 7 days may not be enough to promote the formation of mineralized nodules. Therefore, no calcium nodules in either the TRIB3 interference shRNA group or the negative control shRNA group were detected. These results suggest that TRIB3 may promote differentiation at the middle stage of osteogenic differentiation in hBMSCs.

**Figure 3:**
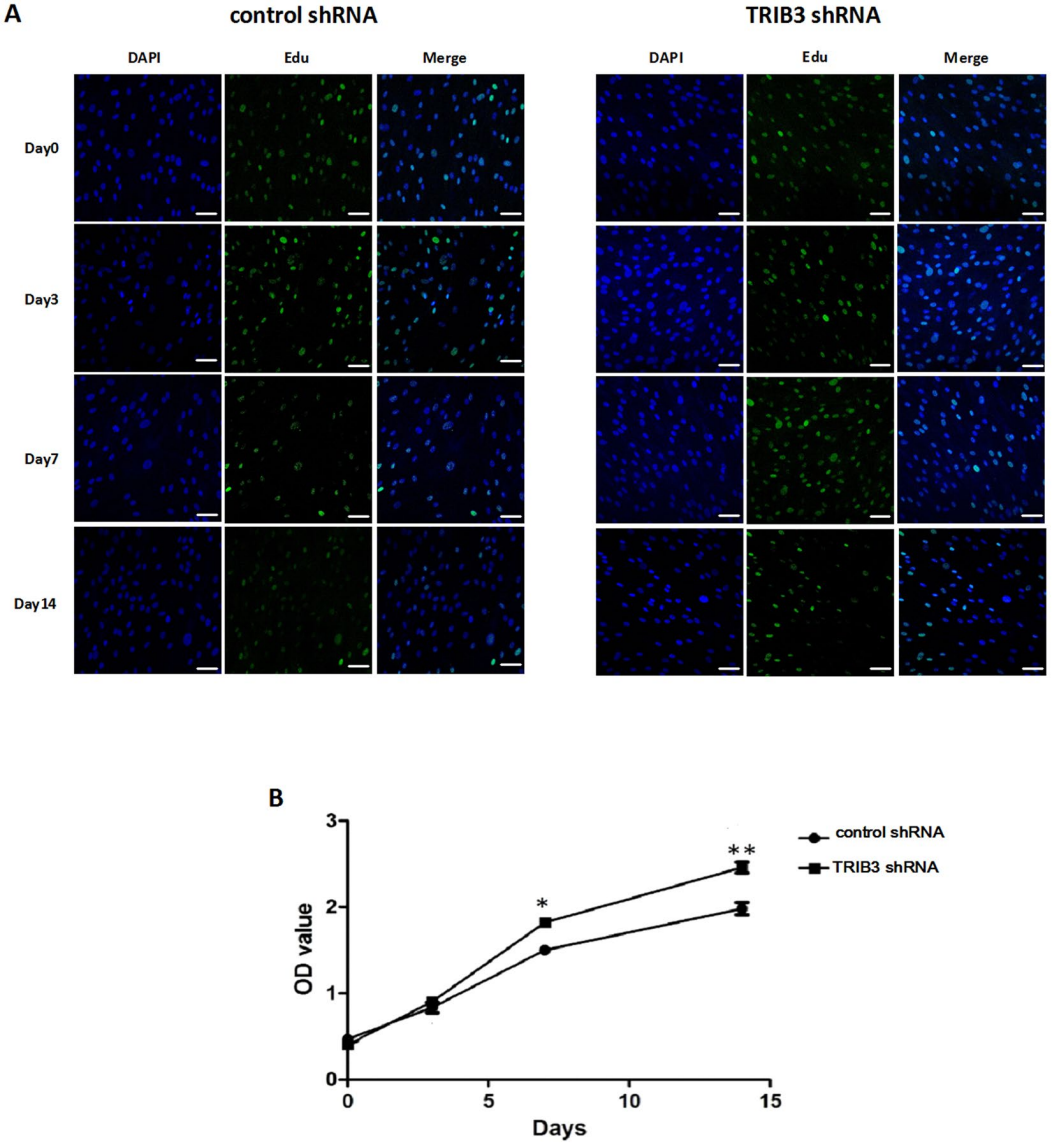
Effects of TRIB3 knock-down on the proliferation of hBMSCs during osteogenic induction. (**A**) Cell proliferation at days 0, 3, 7 and 14 of osteogenic differentiation detected by EdU assay. Green: EdU dye; Blue: DAPI. Scale: 50 μm. (**B**) The proliferation rate from days 0 to 14 of osteogenic differentiation was measured using the CCK8 detection kit. control shRNA: hBMSCs transfected with negative control-shRNA-Vector; TRIB3 shRNA: hBMSCs transfected with TRIB3-shRNA-Vector. *P < 0.05, **P < 0.01.

In analyzing the effects of TRIB3 inhibition on osteogenic differentiation in hBMSCs, we also found that the inhibition of TRIB3 increased cell proliferation. The EdU-labeling assay showed that the proliferation of cells from hBMSCs transfected with TRIB3-shRNA-Vector at day 0 and day 3 of differentiation was unaltered compared with that of cells from hBMSCs and transfected with negative control-shRNA-Vector. However, the proliferation of cells from hBMSCs transfected with TRIB3-shRNA-Vector was higher than that of cells from hBMSCs and transfected with negative control-shRNA-Vector at days 7 and 14 of differentiation (Fig. 3A). Thus, the positive proliferative effect of TRIB3 knock-down was not significant at the early stage but was pronounced at the middle stage of differentiation. We also performed a WST-8 assay to evaluate the effect of TRIB3 knock-down on cell proliferation during osteogenic induction. Figure 3B confirms the role of TRIB3 knock-down in the promotion of cell proliferation. These results indicate that TRIB3 inhibits cell proliferation at the middle stage of osteogenic differentiation.

### FAK regulates the protein expression of TRIB3 in a PI3K/AKT-dependent manner

**Figure 4:**
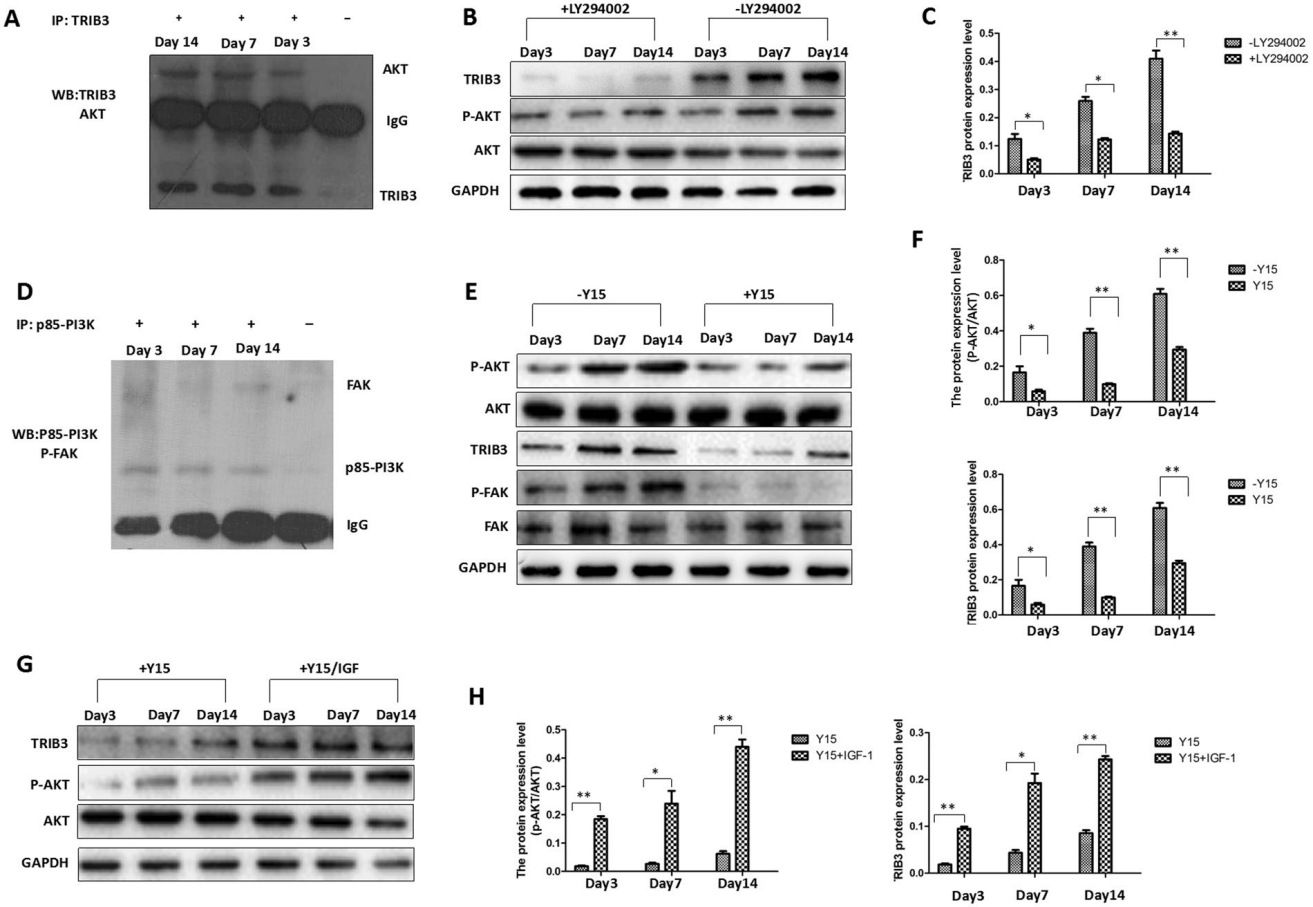
FAK regulates the expression of TRIB3 in a PI3K/AKT-dependent manner. (**A**) Immunoprecipitation assay of cells cultured in the osteogenic induction medium for 3, 7 and 14 days (lines 1–3). Immunoblotting for TRIB3 suggests the success of the immunoprecipitation experiment, and immunoblotting for AKT suggests that AKT can bind to TRIB3 during osteogenic differentiation in hBMSCs. IgG was used as a negative control (line 4). (**B**) Western blot analysis of TRIB3, p-AKT and AKT in cells cultured in the osteogenic induction medium or osteogenic induction medium plus LY294002 for 3, 7, and 14 days. (**C**) The relative activity level of TRIB3 quantified and plotted. (**D**) Immunoprecipitation analysis of cells cultured in the osteogenic induction medium for 3, 7 and 14 days (lines 1–3). Immunoblotting for p85-PI3K suggests the success of the immunoprecipitation experiment, and immunoblotting for FAK suggests that FAK can bind to p85-PI3K during osteogenic differentiation in hBMSCs. IgG was used as a negative control (line 4). (**E**) Western blot analysis of p-FAK, p-AKT, and TRIB3 in cells cultured in the osteogenic induction medium or osteogenic induction medium plus Y15 for 3, 7, and 14 days. (**F**) Relative activity level of p-AKT and TRIB3 quantified and plotted. (**G**) Western blot analysis of p-AKT and TRIB3 in cells cultured in the osteogenic medium plus Y15 or osteogenic medium plus Y15 and IGF-1 for 3, 7, and 14 days. (**H**) Relative activity level of p-AKT and TRIB3 protein quantified and plotted. *P < 0.05, **P < 0.01.

Above results showed the important role of TRIB3 in regulating proliferation and osteogenic differentiation in hBMSCs. However, which signaling pathways regulate the expression of TRIB3 during osteogenic differentiation in hBMSCs? TRIB3 has been identified as a novel transcriptional target of the PI3K/AKT pathway in PC-3 cells^26^. However, the relationship between TRIB3 and the PI3K/AKT pathway in the process of osteogenic differentiation in hBMSCs is unclear. Therefore, an immunoprecipitation assay was performed to determine whether AKT is a binding partner of TRIB3 during osteogenic differentiation. Immunoprecipitation of proteins from cells induced for 3, 7 and 14 days with an anti-TRIB3 antibody revealed a 60-KD band interacting with the anti-AKT antibody (Figs 4A and S3A). Then, we used LY294002 (LY), an inhibitor of PI3K, to examine the role of the PI3K pathway in the expression of TRIB3. hBMSCs were induced for osteogenic differentiation in the presence of LY or solvent dimethyl sulfoxide (DMSO) for 3, 7, or 14 days. The results showed that the activity of AKT, an immediate PI3K effector, was reduced and that the expression of TRIB3 was reduced, which confirmed the successful inhibition of PI3K signaling (Figs 4B and S3B). The decrease in TRIB3 protein expression resulting from LY294002 treatment indicated that the activation of the PI3K/AKT signaling pathway mediated the protein expression of TRIB3 during osteogenic differentiation in hMSCs (Figs 4B,C and S3C).

FAK has been reported to activate the PI3K/AKT pathway^27^. Our immunoprecipitation assay also showed that FAK can directly bind to the p85 subunit of PI3K in cells induced for 3, 7 and 14 days (Figs 4D and S4A), which suggested that FAK may modulate the activation of the PI3K/AKT pathway during osteogenic differentiation in hBMSCs. To this end, we treated cells with Y15, a specific inhibitor of FAK phosphorylation, and DMSO was used as the control (Figs 4E and S4B). Inhibition of FAK activation resulted in a significant decrease in AKT activity (Figs 4E,F and S4C). In addition, TRIB3 protein expression at days 3, 7, and 14 of differentiation was significantly inhibited by Y15 (Figs 4E,F and S4D).

According to the above results, it can be hypothesized that FAK regulates the expression of TRIB3 through the PI3K/AKT signaling pathway. Next, IGF-1 was used to activate PI3K/AKT signaling via the phosphorylation of AKT (Figs 4G,H and S5A). Our results showed that IGF-1 could antagonize the inhibitory effect of Y15 on TRIB3 expression at days 3, 7, and 14 (Figs 4G,H and S5B). Taken together, the increase in FAK activity may promote the expression of TRIB3 in a PI3K/AKT-dependent manner during osteogenic differentiation in hBMSCs.

### TRIB3 inhibits proliferation and promotes osteogenic differentiation in hBMSCs by inhibiting ERK1/2 activity

**Figure 5:**
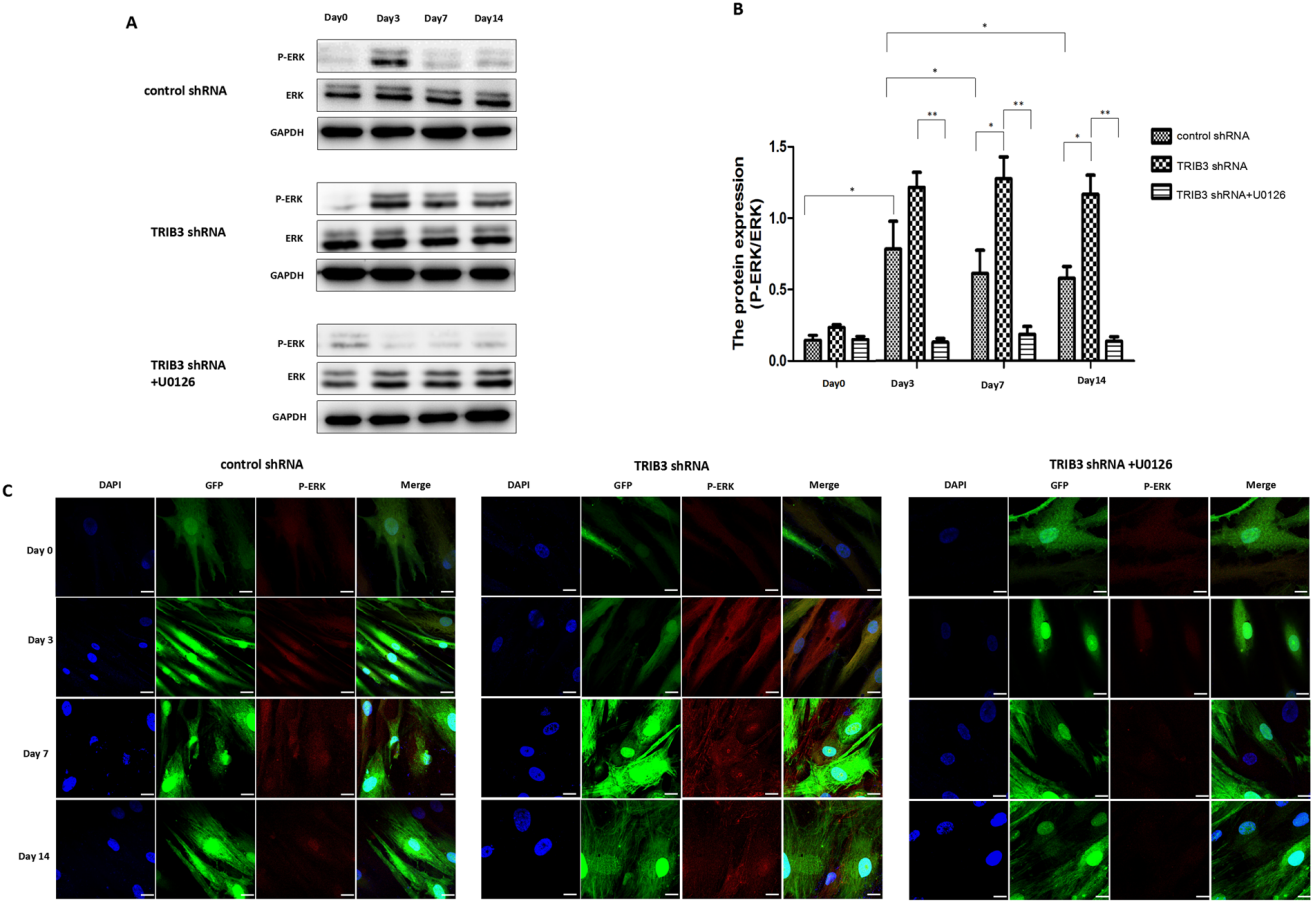
Effects of TRIB3 knock-down on the activity of ERK. (**A**) Western blot analysis of ERK1/2 expression and activity in cells induced for 0, 3, 7, and 14 days. (**B**) Relative activity level of ERK protein quantified and plotted. (**C**) ERK activity measured by immunostaining for anti-p-ERK at days 0, 3, 7, and 14 of osteogenic differentiation. Scale: 40 μm. control shRNA: hBMSCs transfected with negative control-shRNA-Vector; TRIB3 shRNA: hBMSCs transfected with TRIB3-shRNA-Vector; TRIB3 shRNA + U0126: U0126 treatment of hBMSCs transfected with TRIB3-shRNA-Vector. *P < 0.05, **P < 0.01.

Then, the next question is how does TRIB3 regulate proliferation and osteogenic differentiation in hBMSCs. It has been reported that TRIB3 promotes ERK1/2 activity at low concentrations but inhibits ERK1/2 at high concentrations^28^. Therefore, ERK1/2 was presumed to be a possible downstream effector of TRIB3 in osteogenic differentiation in hBMSCs. We examined the expression and activation of ERK1/2 at different stages of osteogenic differentiation. ERK1/2 expression was consistent throughout the osteogenic process. Compared to undiferentiated hBMSCs, cells osteogenically induced for 3 days exhibited a higher level of ERK1/2 phosphorylation. However, a significant decrease in ERK1/2 phosphorylation was observed on days 7 and 14 of differentiation (Figs 5A,B and S6). Thus, ERK1/2 showed a tendency of initial increase, followed by a decrease in its expression at the middle stage of differentiation. Then, the effect of TRIB3 on ERK1/2 activity was evaluated by measuring ERK1/2 phosphorylation in cells induced from hBMSCs and transfected with TRIB3-shRNA-Vector. Immunoblot (Figs 5A,B and S6) and immunofluorescence (Fig. 5C) assays showed that TRIB3 knock-down had no effect on ERK1/2 activity at day 0 and day 3 but increased ERK1/2 activity on days 7 and 14 compared to that in cells induced from control hBMSCs.

**Figure 6:**
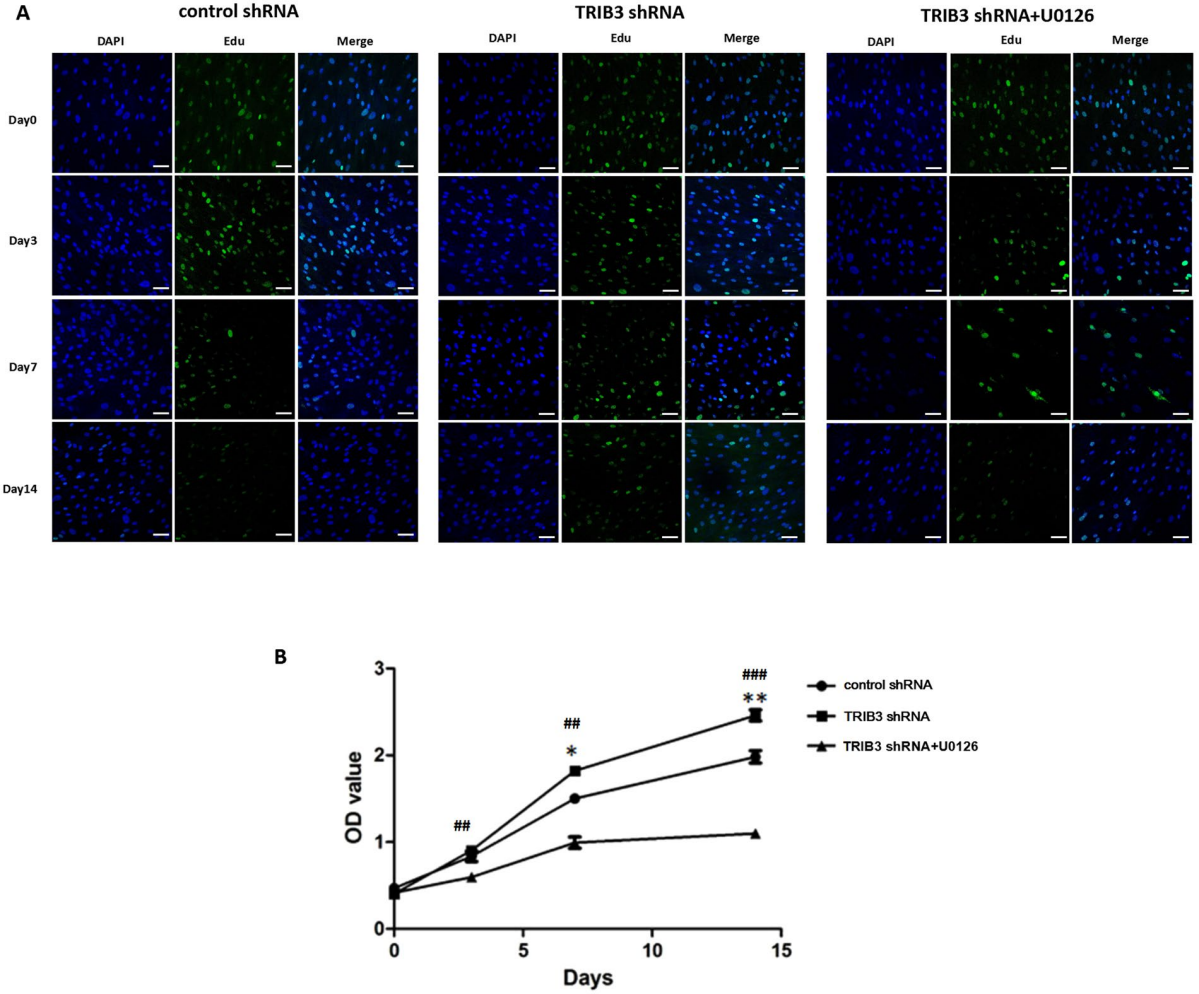
TRIB3 regulates the proliferation of hBMSCs by regulating ERK1/2 activity during osteogenic induction. (**A**) Cell proliferation at days 0, 3, 7 and 14 of osteogenic differentiation measured by EdU assay. Green: EdU dye; Blue: DAPI. Scale: 50 μm. (**B**) The proliferation rate from days 0 to 14 of osteogenic differentiation was measured using the CCK8 detection kit. control shRNA: hBMSCs transfected with negative control-shRNA-Vector; TRIB3 shRNA: hBMSCs transfected with TRIB3-shRNA-Vector; TRIB3 shRNA + U0126: U0126 treatment of hBMSCs transfected with TRIB3-shRNA-Vector. *p < 0.05 and **p < 0.01 used for comparison between TRIB3 shRNA and control shRNA; ^#^p < 0.05, ^##^p < 0.01, ^###^p < 0.005 used for comparison between TRIB3 shRNA and TRIB3 shRNA + U0126.

**Figure 7:**
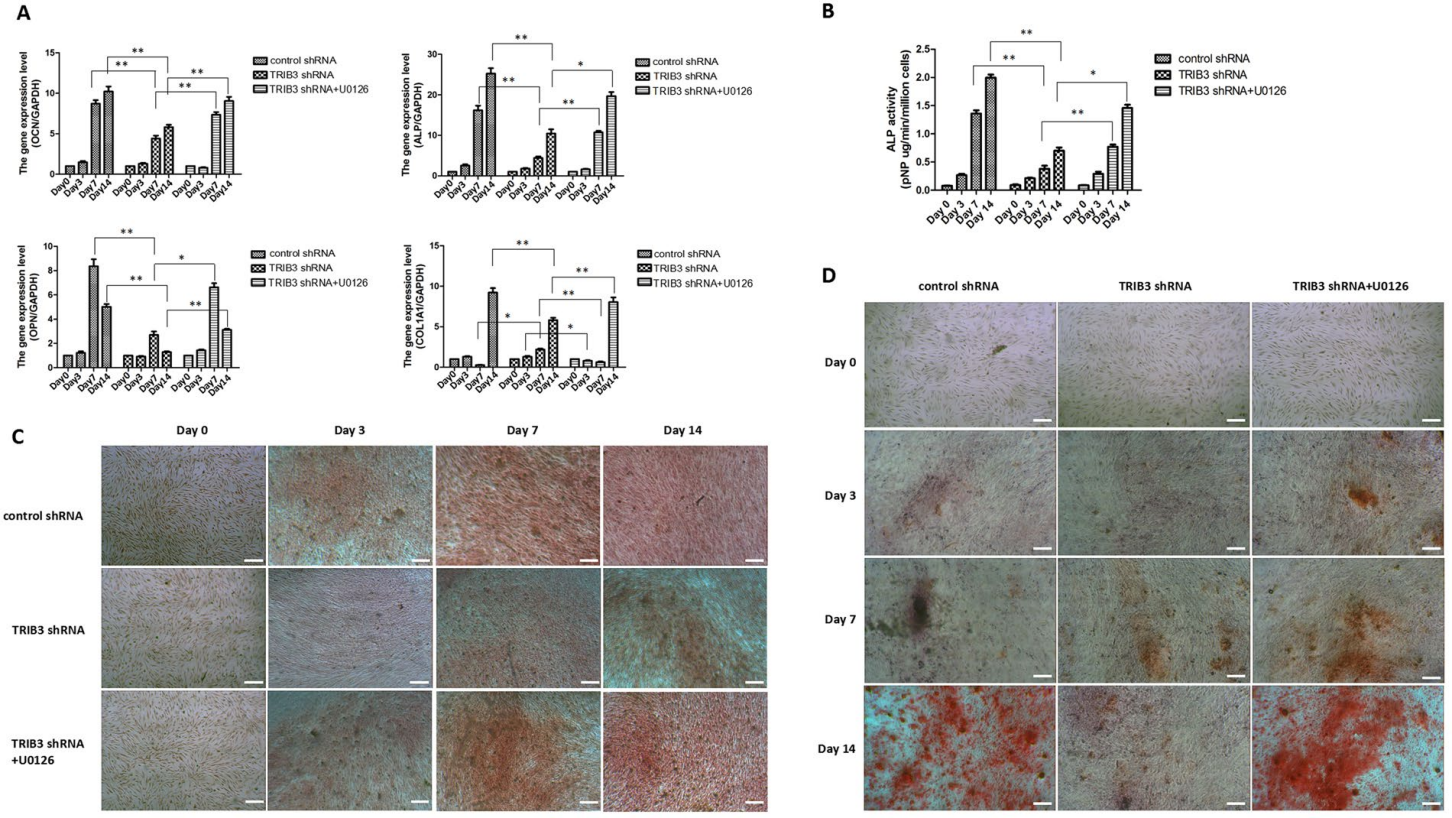
TRIB3 regulates the osteogenesis of hBMSCs by inhibiting ERK1/2 activity during osteogenic induction. (**A**) Real-time PCR analysis of genes specific for osteoblasts, such as *ALP*, *COL1A1*, *OPN*, and *OCN*, in cells induced for 0, 3, 7, and 14 days. (**B**) Analysis of ALP activity in cells induced for 0, 3, 7 and 14 days. (**C**) ALP staining of cells induced for 0, 3, 7 and 14 days. Scale: 100 μm. (**D**) Mineralization (intense red clusters) in cells induced for 0, 3, 7 and 14 days. Scale: 100 μm. control shRNA: hBMSCs transfected with negative control-shRNA-Vector; TRIB3 shRNA: hBMSCs transfected with TRIB3-shRNA-Vector; TRIB3 shRNA + U0126: U0126 treatment of hBMSCs transfected with TRIB3-shRNA-Vector. *P < 0.05, **P < 0.01.

To confirm that ERK1/2 participates in proliferation and osteogenic differentiation in hBMSCs regulated by TRIB3, we used U0126, an ERK-specific inhibitor, to treat hBMSCs transfected with TRIB3-shRNA-Vector. The use of U0126 resulted in a decrease in ERK1/2 activity, which confirmed the successful inhibition of ERK1/2 activity (Figs 5A–C and S6). Then, we used Edu and WST-8 assays to examine the effect of ERK1/2 inhibition on cell proliferation. Figure 6 shows a significant decrease in cell proliferation after adding U0126, especially at days 7 and 14 of differentiation. Moreover, we also investigated whether TRIB3 regulates osteogenic differentiation through the inhibition of ERK1/2 activity. The expression of four osteogenic marker genes (*ALP*, *COL1A1*, *OPN*, and *OCN*) was detected from days 0 to 14 (Fig. 7A). The results showed that the expression of the four marker genes was partly restored at day 7 and day 14 of differentiation by adding U0126 (Fig. 7A). In addition, U0126 treatment increased the ALP activity in cells induced from hBMSCs and transfected with TRIB3-shRNA-Vector for 7 and 14 days but did not affect the ALP activity in cells induced for 0 and 3 days (Fig. 7B and C). Furthermore, the mineralization in cells induced for 14 days was increased by adding U0126 (Fig. 7D). Induction for 0, 3 and 7 days was still too short to form obvious mineralized nodules. Thus, the addition of U0126 can partially restore the inhibitory effect of TRIB3 knock-down on osteogenic differentiation at the middle stage of differentiation. Therefore, it is inferred that TRIB3 inhibits proliferation and promotes osteogenic differentiation in hBMSCs through the inhibition of ERK1/2 activity.

## Discussion

The tribble family is characterized by the evolutionally conserved kinase-like domain that is highly homologous to serine/threonine kinases. In Drosophila, this family includes only one member that plays a role in protein degradation. Three members of the tribble family have been identified in mammals, of which TRIB3 is known to regulate various physiological functions through different signaling pathways, including the canonical TGF-β, MAPK, and Notch signaling pathways^23–25^; these signaling pathways also play a regulatory role in osteogenic differentiation in mesenchymal stem cells (MSCs). In a previous study, it was proven that the downregulation of TRIB3 inhibits BMP-mediated cellular responses, including osteoblast differentiation in C2C12 cells^29^. In the process of characterizing hBMSCs, we found that TRIB3 expression was undetectable in undifferentiated hBMSCs and that TRIB3 expression levels increased with the osteogenic induction time. In addition, TRIB3 has also been reported to inhibit the differentiation of adipocytes^30^. Adipogenesis and osteogenesis in MSCs are antagonistic processes; increased adipogenesis is characterized by the suppression of osteogenesis in MSCs, while the upregulation of osteogenic signaling attenuates adipogenic terminal differentiation^31^. Taking these data together, it is possible that TRIB3 might play a role in osteogenic differentiation in hBMSCs. To verify this possibility, we analyzed the effects of TRIB3 on the osteogenic potential of hBMSCs. ALP is regarded as a transient early marker of osteogenic differentiation^32–34^. Therefore, in the present study, ALP activity was analyzed to determine the early osteogenic differentiation potential of hBMSCs. As shown by our results, ALP activity in cells induced for 7 and 14 days was decreased when TRIB3 expression was inhibited, but no change in ALP activity in cells induced for 0 and 3 days was found. Mineralized nodules are typically observed as a later phase marker for terminal differentiation^35^. Here, we noted the negative effect of the genetic inhibition of TRIB3 on the development of mineralized nodules on day 14 of differentiation. Further observations of the genetic inhibition of TRIB3 indicate a clear reduction in the mRNA expression of osteoblast-specific genes, such as ALP, COL1A1^36, 37^, OCN^38^ and OPN^39^, on days 7 and 14 of differentiation. These results verify our proposal that TRIB3 plays an important role in osteogenic differentiation in hBMSCs.

It is now well established that the osteogenic differentiation of MSCs is marked by sequential stages of cellular proliferation and bone extracellular matrix maturation^40^. As the inhibition of TRIB3 expression suppressed osteogenic differentiation, we thus investigated whether TRIB3 expression resulted in growth arrest in cells during osteogenic differentiation. The inhibition of TRIB3 expression enhanced cell proliferation at days 7 and 14 of osteogenic differentiation compared to that of controls. Our results appear to be consistent with previous studies showing that tribbles inhibit cell proliferation in Drosophila and human cells^41–43^. Interestingly, the inhibition of TRIB3 expression did not affect the differentiation or cell proliferation at day 0 and 3 of osteogenesis. After further analysis, we observed that TRIB3 was expressed throughout early and middle osteogenic differentiation, and its expression level was higher at the middle stage (days 7 and 14) compared to that in the early stage (day 0 and 3) of osteogenic differentiation. Therefore, it may be suggested that the high expression of TRIB3 inhibits the proliferation of hBMSCs and promotes differentiation in the middle stage of osteogenic differentiation; however, low expression of TRIB3 at the early stage has no obvious role in proliferation and differentiation.

ERK1/2 is a downstream component of the MAPK/ERK pathway and is thought to promote proliferation in hBMSCs. However, its role in osteogenic differentiation is controversial. While some reports suggest that ERK1/2 promotes osteogenic differentiation in MSCs, others show that it inhibits osteogenic differentiation^44–48^. Additionally, some studies show that ERK1/2 mediates cell proliferation following its transcriptional activation by certain factors^49–51^. A previous report showing that TRIB3 promotes ERK1/2 activity at low concentrations but inhibits ERK1/2 at high concentrations reveals the possibility that TRIB3 plays a role in proliferation and osteogenic differentiation in hBMSCs^28^. In the present study, TRIB3 knock-down did not affect the activity of ERK1/2 on day 0 and 3 but promoted the activity of ERK1/2 on days 7 and 14 of osteogenic differentiation. Because the high expression of TRIB3 was associated with the middle stage and the low expression of TRIB3 was associated with the early stage of osteogenic differentiation, we believe that the high expression of TRIB3 at the middle stage inhibited ERK1/2 activity; yet, the low expression of TRIB3 at the early stage had no effect on ERK1/2 activity. To further determine whether ERK1/2 is involved in cell proliferation and osteogenesis regulated by TRIB3, we treated cells induced from hBMSCs and transfected with TRIB3-shRNA-Vector with the ERK1/2 inhibitor U0126. The results showed that the inhibitory effect of TRIB3 knock-down on osteogenesis differentiation was partly restored and that proliferation was decreased at the middle stage of osteogenic differentiation. These results suggest that TRIB3 inhibits proliferation and promotes osteogenic differentiation in hBMSCs by decreasing ERK1/2 activity at the middle stage of osteogenic differentiation.

The osteogenic differentiation of MSCs is a complex process orchestrated by multiple signaling pathways. In mice, there is substantial evidence that PI3K/AKT signaling is important for osteogenesis and endochondral ossification^52^. In cultures of 2T3 osteoblast precursor cells isolated from mouse calvaria, BMP-induced osteogenesis requires PI3K/AKT signaling^53^. AKT activity is also required for the growth of isolated mouse metatarsal bones *in vitro* during the chondrogenic phase of the process and for osteoblast maturation^54^. In humans, the PI3K/AKT pathway is important for osteogenesis. Recent studies using human MSCs revealed the importance of the PI3K/AKT pathway in promoting osteoblast differentiation^55^. A previous study reported that in PC-3 cells, decreasing AKT-phosphorylation by using LY094002 or knocking down p110β inhibited TRIB3 expression^26^. Using an immunoprecipitation assay, we found that TRIB3 can directly bind to AKT, which is consistent with a previous report^17^. Therefore, we hypothesized that the expression of TRIB3 was also regulated by AKT-phosphorylation during osteogenesis in hBMSCs. The treatment of cells induced for osteogenic differentiation with the PI3K inhibitor LY caused decreased TRIB3 expression, highlighting that TRIB3 expression during osteogenic differentiation is dependent on AKT activity. Our results are contradictory to a previous report showing that TRIB3 can directly bind to AKT and act as a suppressor of AKT by inhibiting phosphorylation in liver cells^17^. This difference may be due to the use of different cells and physiological functions. In addition, the mechanism for the interaction between TRIB3 and AKT proteins is different, and the specific mechanism requires further study.

Focal adhesion kinase (FAK) is involved in regulating ECM-induced osteogenic differentiation in MSCs. FAK inhibition decreased the serine phosphorylation of Runx2/Cbfa-1 and osterix transcriptional activity, as well as the osteogenic differentiation of MSCs^7^. In this study, we found that inhibiting FAK activity by using Y15 resulted in a significant decrease in the expression of TRIB3 during the early and middle stages of osteogenic differentiation, indicating a positive effect of FAK activation on TRIB3 expression during osteogenic differentiation in hBMSCs. It has been reported that the ligation and clustering of integrins activate FAK by autophosphorylating tyrosine 397, creating a potential binding site for the SH2 domains of the p85 subunit of PI3K^56, 57^. Phosphorylation of the p85 subunit of PI3K by FAK may activate the p110 catalytic subunit of PI3K and thereby activate the PI3K/AKT signaling pathway. We also found that FAK can directly bind to the p85 subunit of PI3K. Additionally, we found a decrease in AKT activity following the inhibition of FAK phosphorylation during osteogenic differentiation. Together with the above data, we speculate that activated FAK may regulate TRIB3 protein expression through activating the PI3K/AKT pathway. To verify this hypothesis, IGF-1 was used to activate PI3K/AKT signaling in Y15-treated cells, which restored the protein expression of TRIB3. In the early and middle stages of osteogenic differentiation in hBMSCs, activated FAK can bind PI3K and thus activate the PI3K/AKT pathway; then, activated AKT can bind TRIB3 and promote its expression.

In summary, the present study presents evidence that the activated FAK/PI3K/AKT pathway regulates the expression of TRIB3, while TRIB3 promotes osteogenesis and inhibits cell proliferation at the middle stage of osteogenic differentiation in hBMSCs. These effects are evident at the middle stage of osteogenesis partly because of the inhibition of ERK1/2 activity.

## Materials and Methods

### Preparation of hBMSCs and osteogenic induction

This study was performed in accordance with standard ethical guidelines and approved by the Institutional Review Board (IRB) of Zhejiang University, Hangzhou, China, and the Ethical Committee of the First Affiliate Hospital, Zhejiang University, China. After obtaining informed consent from all donors, whole-bone marrow samples were collected at the First Affiliate Hospital, Zhejiang University, from three healthy donors who were 19 (female), 23 (male) and 34 (female) years old. hBMSCs were isolated from the bone marrow as follows: 4 mL of bone marrow was gently flushed with 40 mL of phosphate-buffered saline (PBS) supplemented with 1% heat-inactivated fetal bovine serum (FBS; Gibco BRL, Shanghai, China). Marrow cells were pelleted by centrifugation at 1,800 rpm for 5 min at room temperature. The fat-containing supernatant was removed, and the pellet was resuspended in 6 mL of PBS supplemented with 1% FBS. The pellet was purified by density gradient centrifugation using 4.5 mL of lymphocyte separation medium (LSM; MP Biomedicals, Shanghai, China), i.e., the suspension was divided into three layers by centrifugation at 500 rpm for 25 min at room temperature. Cells at the interface were harvested, washed with PBS containing 1% FBS, and seeded in a 10-cm dish containing α-MEM culture medium (Gibco, Shanghai, China) supplemented with 10% FBS, 100 U/mL penicillin (Gibco), 100 μg/mL streptomycin (Gibco), and 5 ng/mL basic fibroblast growth factor (BFGF; Life Technologies, Shanghai, China). Cells were cultured under standard conditions at 37 °C and 5% carbon dioxide (CO_2_) and 95% humidity. Confluent cells were trypsinized and reseeded at a density of 1 × 10^5^ cells/mL until passage, and cells at passage 3 were used for the experiments.

Before the induction experiment, cells at passage 3 were examined for the expression profile of special surface antigens. The cells prepared from human bone-marrow expressed CD19^−^, CD34^−^, CD14^−^, CD45^−^, HLA-DR^−^, CD105^+^, CD90^+^, CD73^+^ and CD29^+^ (Fig. S7). In addition, these cells had osteogenic and adipogenic potential (Fig. S7). These characteristics indicate that the cells prepared from human bone marrow can be accepted as hBMSCs^58^.

When the cells reached 60–70% confluency at passage 3, the medium was replaced with osteogenic medium^59^ (L-Dulbecco’s modified Eagle’s medium (DMEM; Life Technologies, Shanghai, China), 10% FBS, 50 μg/mL L-ascorbic acid (Sigma, Shanghai, China), 10 mM β-glycerophosphate (Sigma), 0.1 μM dexamethasone (Sigma), 100 U/mL penicillin, and 100 μg/mL streptomycin). We selected four time points of induction to perform various analyses to study the early and middle stages of osteogenic differentiation; day 0 and day 3 of osteogenic differentiation were designated as the early stage, and day 7 and day 14 of osteogenic differentiation were designated as the middle stage. The subsequent results follow this definition unless otherwise specified.

### 5-Ethynyl-2′-deoxyuridine (EdU) labeling

5-Ethynyl-2′-deoxyuridine labeling was performed according to the manufacturer’s protocol (RiboBio, Guangzhou, China). Briefly, hBMSCs were cultured in 24-well plates at 3 × 10^4^ cells/well for 24 h at 37 °C; then, the culture medium was replaced with osteogenic medium. hBMSCs cultured in osteogenic medium for 0, 3, 7, and 14 days were treated with 50 μM EdU for 2 h at 37 °C. Following incubation, the cells were fixed with 4% formaldehyde for 15 min and permeabilized with 0.5% Triton X-100 for 20 min at room temperature. The cells were washed thrice with PBS and incubated with 100 μL of 1 × Apollo reaction cocktail for 30 min at room temperature. After incubation, cells were stained with 100 μL of 4′, 6-diamidino-2-phenylindole (DAPI) for 3 min and visualized under a Zeiss confocal microscope. All experiments were performed in triplicate, and three independent experiments were performed^60^.

### Cell proliferation assay

Cell proliferation was evaluated using the WST-8 (4-[3-(2-methoxy-4-nitrophenyl)-2- (4-nitrophenyl)-2H-5-tetrazolio]-1, 3-benzene disulfonate sodium salt; Cell Counting Kit-8, Qi Hai, Hangzhou, China) assay at days 0, 3, 7, and 14 of osteogenic induction. Cells were seeded into 96-well plates at 7.5 × 10^3^ cells/well in 200 μL of medium. The WST-8 solution was added to the culture medium, and the cells were incubated at 37 °C for an additional 1.5 h. The absorbance of the reaction solution was measured at wavelength of 450 nm^61^. All experiments were performed in quintuplicate, and three independent experiments were performed.

### Real-time (reverse transcriptase) polymerase chain reaction (PCR) assays

We determined the expression of TRIB3 and osteogenesis-specific genes by PCR. RNA extraction was performed using the TRIzol reagent (Sigma). Reverse transcription was performed using 1 μg of total RNA and the RevertAid First Strand cDNA Synthesis Kit (Fermentas, Shanghai, China). Primers were designed using Primer Premier 6.0 Demo and Oligo 7.36 Demo software as shown in Table 1. Quantitative real-time PCR reactions were performed in triplicate on a Roche 480 II Real-Time PCR Detection System using the LightCycler^®^ 480 SYBR Green qPCR Supermix (Roche, Shanghai, China). The PCR conditions were as follows: 40 cycles at 95 °C for 15 s and 60 °C for 1 min^62^. Glyceraldehyde 3-phosphate dehydrogenase (GAPDH) served as the reference gene. All experiments were performed in triplicate, and three independent experiments were performed.

**Table 1:** Primer sequences for real time-PCR.

Gene name	Primer sequences	Product size(bp)
ALPL	5′-AACATCAGGGACATTGACGTG-3′(F)	159
	5′-GTATCTCGGTTTGAAGCTCTTCC-3′(R)	
COL1A1	5′-GTGCGATGACGTGATCTGTGA-3′(F)	119
	5′-CGGTGGTTTCTTGGTCGGT-3′(R)	
OPN	5′-CTCCATTGACTCGAACGACTC-3′(F)	230
	5′-CAGGTCTGCGAAACTTCTTAGAT-3′(R)	
OCN	5′-GGCGCTACCTGTATCAATGG-3′(F)	110
	5′-GTGGTCAGCCAACTCGTCA-3′(R)	
TRIB3	5′-GCCTTTTTCACTCGGACCCAT-3′(F)	172
	5′-CAGCGAAGACAAAGCGACAC-3′(R)	
GAPDH	5′-GGAGCGAGATCCCTCCAAAAT-3′(F)	197
	5′-GGCTGTTGTCATACTTCTCATGG-3′(R)	

### Whole-cell protein extraction and western blot analysis

Cell lysates were prepared by sonication with ice-cold lysis buffer containing 1 mM sodium fluoride (NaF), 1% Nonidet P-40, 0.25% sodium deoxycholate, 1 mM sodium orthovanadate (Na_3_VO_4_), 1 mM ethylenediaminetetraacetic acid (EDTA), 150 mM sodium chloride (NaCl), 1 mM phenylmethylsulfonyl fluoride (PMSF), and a phosphatase inhibitor. Protein concentrations were determined using a bovine serum albumin (BSA) protein assay kit (Beytone, Shanghai, China). Expression levels of TRIB3, FAK, p-FAK, AKT, p-AKT, ERK1/2, p-ERK1/2, and GAPDH were determined by western blot analysis. Proteins from each sample were separated on a 10% sodium dodecyl sulfate-polyacrylamide gel electrophoresis (SDS-PAGE) gel and transferred onto a 4.5 μm polyvinylidene difluoride (PVDF) membrane (Millipore, Shanghai, China). The membrane was blocked using a BLOTTO solution and incubated with anti-ERK1/2, anti-p-ERK1/2, anti-FAK, anti-p-FAK, anti-AKT, anti-p-AKT, anti-GAPDH (Cell Signaling Technology, Shanghai, China) and anti-TRIB3 (Abcam, Shanghai, China) antibodies at 4 °C overnight. Following incubation, the membrane was reprobed with the appropriate secondary antibodies (conjugated with horseradish peroxidase) for 1 h. Protein bands were visualized using the enhanced chemiluminescence detection reagent (Thermo Scientific, Shanghai, China) and a Tanon 6600 Luminescence Imaging Workstation (Tanon, China). All experiments were performed in triplicate, and three independent experiments were performed.

### Immunoprecipitation

Total protein extraction was performed according to the instructions of the Total Protein Extraction Kit for Animal Cultured Cells and Tissue User Manual v4 (Invent Biotechnologies, Jiangsu, China). Native total protein extraction lysis buffer (lysis buffer, 1 mM PMSF and a phosphatase inhibitor) was added to the cell culture plates, and the plates were placed on ice for 5 min. The lysate was collected and centrifuged at 14000 rpm for 30 sec at 4 °C. The supernatant was transferred to new tubes, and anti-TRIB3 or anti-PI3K or anti-IgG (Cell Signaling Technology, Shanghai, China) antibodies were added to the tubes. After rotating for 2 h at 4 °C, 50 μl of protein A/G beads (GenScript, Jiangsu, China) was added to each tube. The tubes were then rotated for 1 h at room temperature. After detachment from the beads by adding loading buffer and boiling at 100 °C, the proteins were separated by SDS-PAGE and analyzed by western blotting as described above. Anti-IgG antibody was used as a negative control. All experiments were performed in triplicate, and three independent repeating experiments were performed.

### Immunocytochemistry

Cells cultured on cover slips were fixed with 4% paraformaldehyde for 15 min, followed by permeabilization with 0.5% Triton X-100 for 10 min at room temperature. The cells were blocked with PBST (PBS with Tween-20) containing 1% BSA and incubated with anti-TRIB3 and anti-p-ERK antibodies at 4 °C overnight. Following incubation, the cover slips were reprobed with the appropriate secondary antibodies for 1 h. The cells were stained with 100 μL of DAPI for 3 min, and images were obtained using a Zeiss confocal microscope. Cells were counted as positive for green or red fluorescence when the fluorescent intensity was greater than the 99th intensity percentile point in channel 2 (green) or 3 (red). All experiments were performed in triplicate, and three independent experiments were performed.

### Alkaline phosphatase (ALP) functional assay

The analysis of ALP activity and ALP staining was performed using cells osteogenically induced for 0, 3, 7 and 14 days. ALP activity was analyzed using the ALP Quantitative Analysis Kit (Nanjing Jiancheng Institute, China), and ALP distribution was analyzed using the Gomori Staining Kit (Nanjing Jiancheng Institute, China) according to the manufacturers’ instructions. All experiments were performed in triplicate, and three independent experiments were performed.

### Alizarin red S (ARS) mineralization assay

Cell osteogenically induced for 0, 3, 7 and 14 days were stained with ARS to investigate the matrix concentrations of calcium and phosphate. ARS was performed according to previously described procedures^63^. Cells were washed thrice with PBS and fixed in ice-cold 95% ethanol for 10 min. Cells were incubated with a 0.4% ARS solution in water (pH 4.2) for 30 min at 37 °C, followed by thorough washing (thrice) with distilled water. Cellular specimens were scored according to the quantity and size of precipitated granules. All experiments were performed in triplicate, and three independent experiments were performed.

### shRNA-mediated knock-down of TRIB3

shRNA corresponding to human TRIB3 mRNA (sense: 5′-GCG GTT GGA GTT GGA TGA CAA-3′ and antisense: 5′-TTG TCA TCC AAC TCC AAC CGC-3′) was designed. Another shRNA (sense: 5′-GAA CGG CAT CAA GGT GAA C-3′ and antisense: 5′-GTT CAC CTT GAT GCC GTT C-3′) was designed as a negative control. shRNA fragments were ligated to the pRNAi-U6.2/Lenti vector, and target plasmids, as well as lentiviral packaging plasmids, were cotransfected into 293 T cells by using Lipofectamine™ 3000 (Invitrogen, Shanghai, China) according to the manufacturer’s instructions. After 48 and 72 h, the virus supernatant was collected and used to infect hBMSCs. Cells transfected with the plasmid exhibited GFP expression. The infection efficiency was detected by real-time PCR, western blot analysis and immunocytochemistry. All experiments were performed in triplicate, and three independent repeating experiments were performed.

### Statistical analyses

Western blot images were semi-quantitatively analyzed with ImageJ software (NIH, USA). The results are expressed as the mean ± standard error of the mean (SEM). Statistical significance was determined using Student’s *t*-test or two-way analysis of variance followed by post hoc Bonferroni correction (p < 0.05).

## Electronic supplementary material

Supplement material
