# Supplementary material for: TRIB3 inhibits proliferation and promotes osteogenesis in hBMSCs by regulating the ERK1/2 signaling pathway
Source: Sci Rep. 2017 Sep 4;7:10342. doi: 10.1038/s41598-017-10601-w (PMC5583332; doi:10.1038/s41598-017-10601-w)
Supplement: Supplementary file 1 — Supplement material [file 41598_2017_10601_MOESM1_ESM.pdf]

Supplementary Material

TRIB3 inhibits proliferation and promotes osteogenesis in hBMSCs by regulating the ERK1/2 signaling pathway

Cui Zhang[1], Fan-Fan Hong[1], Cui-Cui Wang[1], Liang Li[1], Jian-Ling Chen[1], Fei Liu[2], Ren-Fu Quan[2]*, Jin-Fu Wang[1]*

1. Institute of Cell and Development Biology, College of Life Sciences, Zijingang Campus, Zhejiang University, Hangzhou, Zhejiang 310058, P. R. China
2. Institute of Orthopedics, Xiaoshan Traditional Chinese Medical Hospital, Hangzhou, Zhejiang 311200, P. R. China

* Corresponding author: Jin-Fu Wang, College of Life Sciences, Zijingang Campus, Zhejiang University, number 866 of Yuhangtang road, West Lake Dist, Hangzhou, Zhejiang Province, P.R. China. E-mail: wjfu@zju.edu.cn; Ren-Fu Quan, Institute of Orthopedics, Xiaoshan Traditional Chinese Medical Hospital, Hangzhou, Zhejiang 311201, China. E-mail: quanrenfu@126.com.

Figure S1

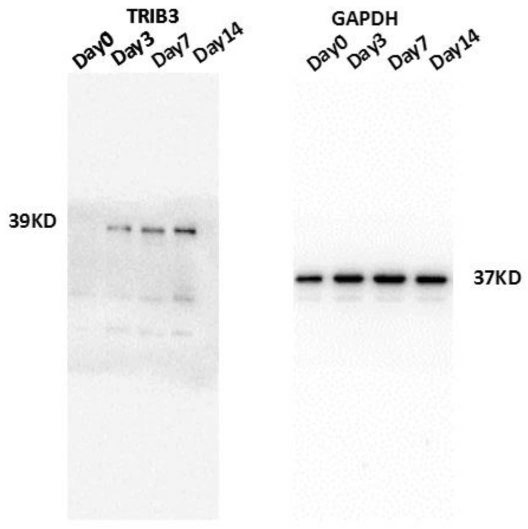

Figure S1: Western blot analysis of TRIB3 protein expression in cells induced for 0, 3, 7, and 14 days. The molecular weight of TRIB3 protein was 39KD. GAPDH was used as loading control.

Figure S2

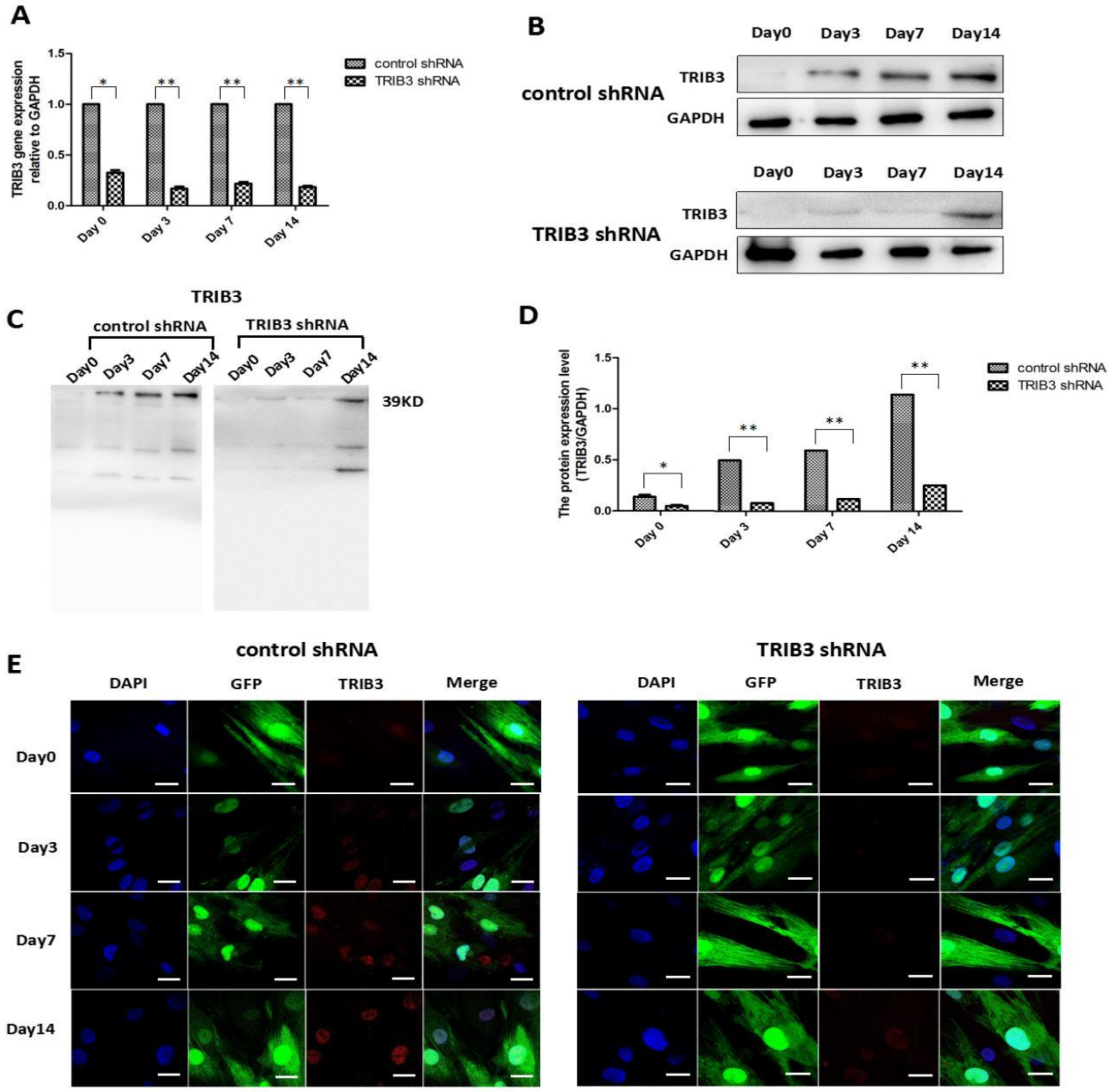

Figure S2. TRIB3 knocked-down by shRNA-interference. (A) Real-time PCR analysis of TRIB3 mRNA expression in cells induced for 0，3, 7, and 14 days. (B) Western blot analysis of TRIB3 protein expression in cells induced for 0， 3, 7, and 14 days. (C) No cropped picture of western blot analysis of TRIB3 protein expression in cells induced for 0， 3, 7, and 14 days. The molecular weight of TRIB3 protein was 39KD. (D) The relative expression level of TRIB3 protein quantified and plotted. (E) Expression of TRIB3 protein revealed by immunostaining with anti-TRIB3 at day 0, 3, 7, and 14 of osteogenic differentiation. Green: GFP on the interfering plasmid; Red: TRIB3; Blue: DAPI. Scale: 40 μm. control shRNA: hBMSCs transfected with negative control-shRNA-Vector; TRIB3 shRNA: hBMSCs transfected with TRIB3-shRNA-Vector. *P<0.05, **P<0.01.

Figure S3

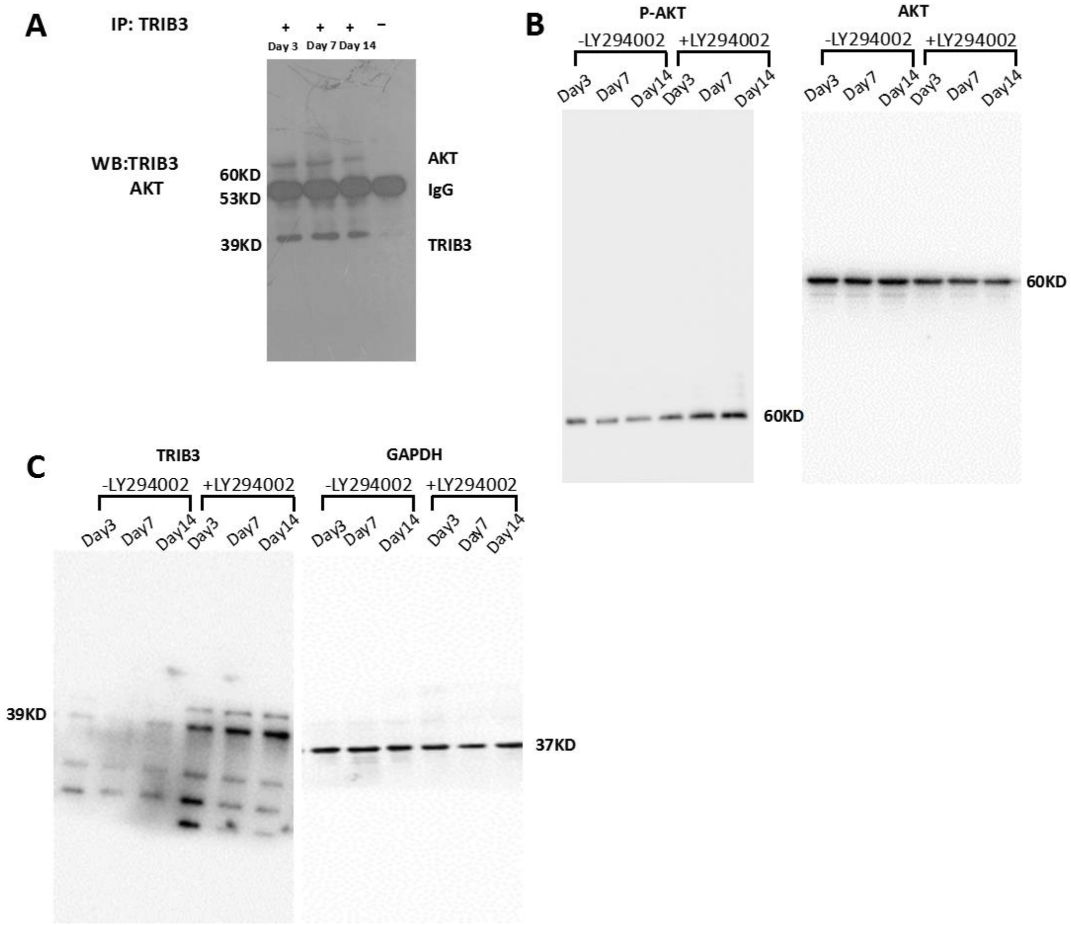

Figure S3: Immunoprecipitation and western blot analysis of protein expression. (A) Immunoprecipitation analysis of cells cultured in the osteogenic induction medium for 3, 7 and 14 days (lines 1-3). Immunoblotting against TRIB3 suggests a success of immunoprecipitation experiment and immunoblotting against AKT suggests that AKT can bind to TRIB3 during osteogenic differentiation of hBMSCs. IgG as a negative control (line 4). (B) Western blot analysis of P-AKT and AKT protein expression in cells induced for 3, 7, and 14 days. (C) Western blot analysis of TRIB3 protein expression in cells induced for 3, 7, and 14 days. GAPDH was used as loading control. -LY094002: cells cultured only in osteogenic medium, +LY094002: cells cultured in both osteogenic medium and LY094002.

Figure S4

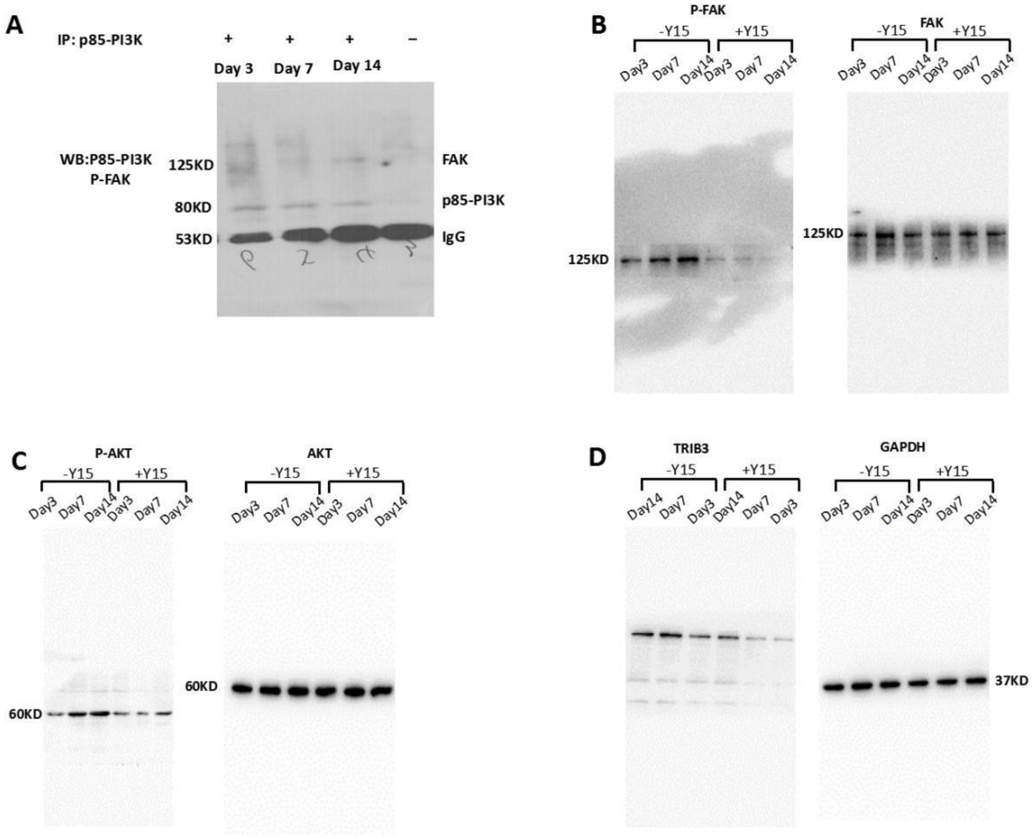

Figure S4: Imunoprecipitation and western blot analysis of protein expression. (A) Immunoprecipitation analysis of cells cultured in the osteogenic induction medium for 3, 7 and 14 days (lines 1-3). Immunoblotting against p85-PI3K suggests a success of immunoprecipitation experiment and immunoblotting against FAK suggests that FAK can bind to p85-PI3K during osteogenic differentiation of hBMSCs. IgG as a negative control (line 4). (B) Western blot analysis of P-FAK and FAK protein expression in cells induced for 3, 7, and 14 days. (C) Western blot analysis of p-AKT and AKT protein expression in cells induced for 3, 7, and 14 days. (D) Western blot analysis of TRIB3 protein expression in cells induced for 3, 7, and 14 days. GAPDH was used as loading control. –Y15: cells cultured only in osteogenic medium, +Y15: cells cultured in both osteogenic medium and Y15.

Figure S5

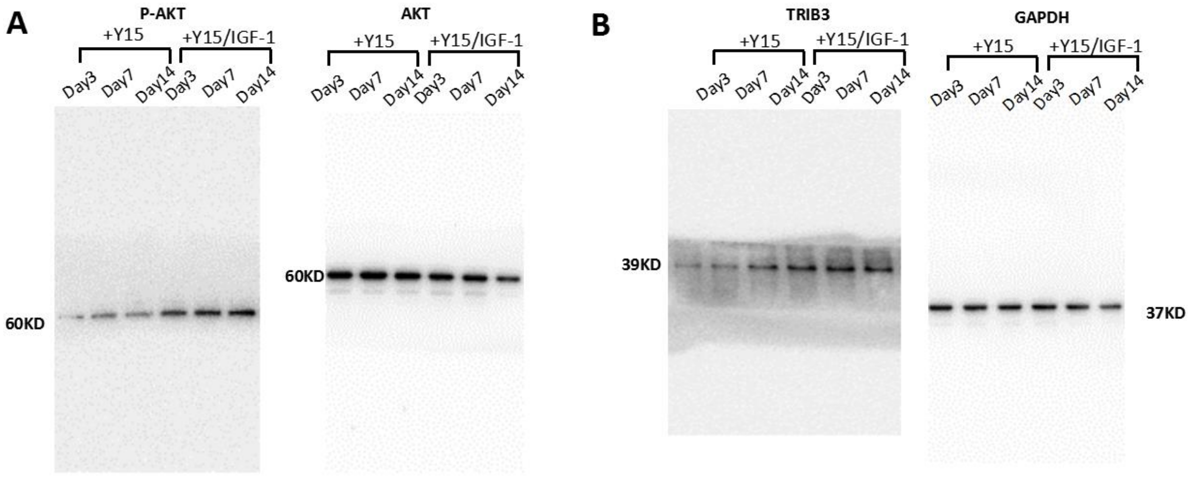

Figure S5: Western blot analysis of protein expression. Cells cultured in the osteogenic medium plus Y15 (+Y15) or osteogenic medium plus Y15 and IGF-1 (+Y15/IGF-1) for 3, 7, and 14 days. (A) Western blot analysis of P-AKT and AKT protein expression in cells induced for 3, 7, and 14 days. (B) Western blot analysis of TRIB3 protein expression in cells induced for 3, 7, and 14 days. GAPDH was used as loading control.

Figure S6

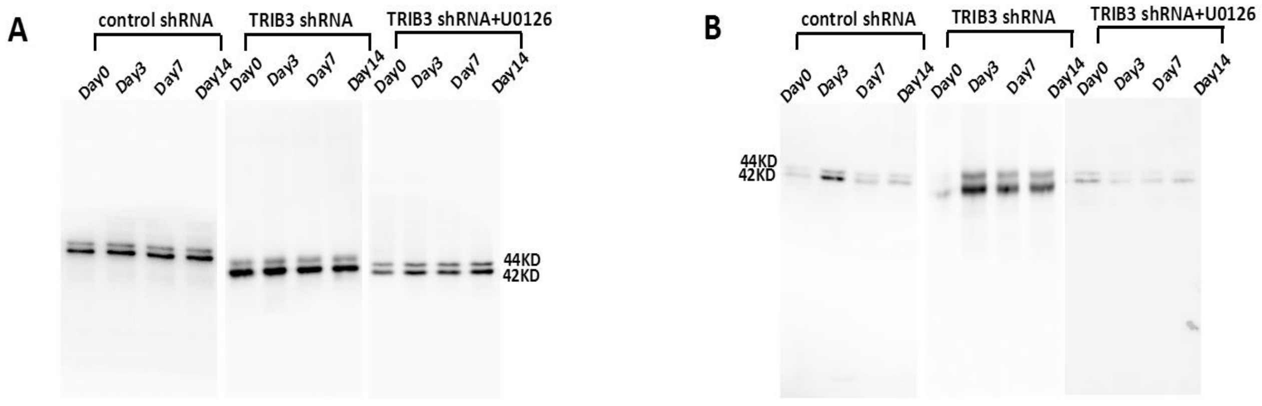

Figure S6: Western blot analysis of ERK and P-ERK expression. (A) Western blot analysis of ERK1/2 protein expression in cells induced for 0, 3, 7, and 14 days. (B) Western blot analysis of ERK1/2 protein activity (P-ERK1/2) in cells induced for 0, 3, 7, and 14 days. control shRNA: hBMSCs transfected with negative control-shRNA-Vector; TRIB3 shRNA: hBMSCs transfected with TRIB3-shRNA-Vector; TRIB3 shRNA+U0126: U0126 treatment for hBMSCs transfected with TRIB3-shRNA-Vector.

Figure S7

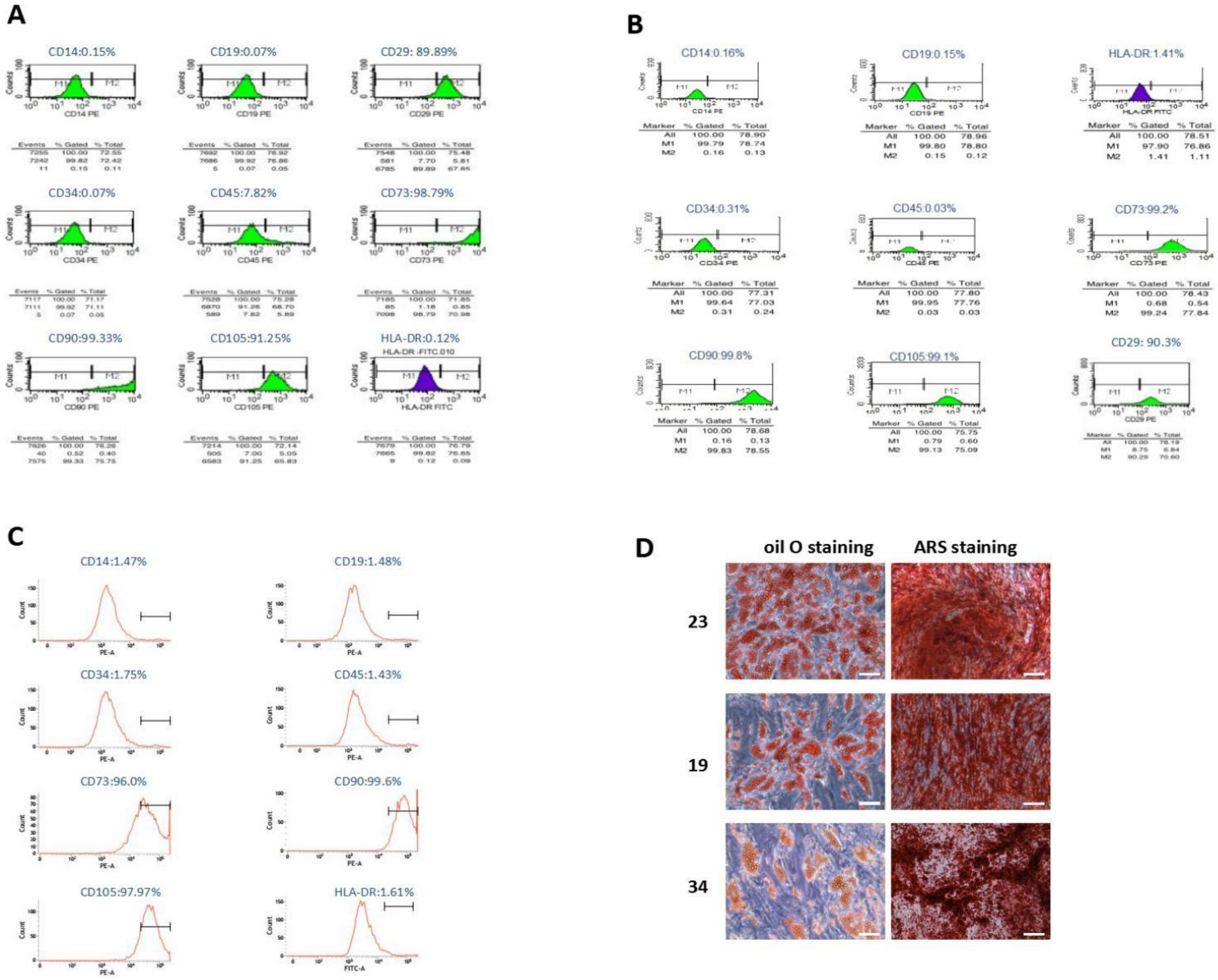

Figure S7 : Analysis of surface markers and differentiation potentials of hBMSCs. (A) Flow cytometric analysis of surface markers in hBMSCs isolated from 23-year-old man showed positive for CD29, CD73, CD90, and CD105 and negative for CD34, CD19, CD45, CD14, and HLA-DR. (B) Flow cytometric analysis of surface markers in hBMSCs isolated from 19-year-old woman showed positive for CD29, CD73, CD90, and CD105 and negative for CD34, CD19, CD45, CD14, and HLA-DR. (C) Flow cytometric analysis of surface markers in hBMSCs isolated from 34-year-old man showed positive for CD73, CD90, and CD105 and negative for CD34, CD19, CD45, CD14, and HLA-DR. (D) The osteogenic and adipogenic potentials of hBMSCs isolated from 23-year-old man, 19-year-old woman and 34-year-old woman were evaluated by ARS and Oil Red O staining. Scale: 40 μm.
